# Underdiagnosed Complicated *Plasmodium vivax* Malaria in a Child: Need to Think

**Published:** 2017

**Authors:** Sonu AGRAWAL

**Affiliations:** Dept. of Microbiology, All India Institute of Medical Sciences, Newdelhi, India

**Keywords:** Malaria, *Plasmodium vivax*, Anemia, Altered sensorium

## Abstract

Malaria is major cause of morbidity and mortality worldwide. The highest incidence of malaria in the world is South East Asia. India is most affected country followed by Indonesia and Myanmar. The prevalence of malaria varies according to geographical region. Diagnosis of malaria infection has been underestimated. Here, we report a case of under-diagnosed complicated *vivax* malaria in one-yr-old child in 2016. Early diagnosis and prompt treatment can reduce both morbidity and mortality in complicated malaria infection.

## Introduction

Malaria as a parasitic disease is endemic in more than 90 countries (1). Malaria is major cause of morbidity and mortality worldwide and transmitted by bite of female anopheles mosquitoes, congenitally and transfusion of blood. The highest incidence of malaria in the world is South East Asia. India is the most affected country followed by Indonesia and Myanmar. The prevalence of malaria varies according to geographical region (2).

The first transfusion transmitted malaria was reported in 1911(3–5). Transfusion of blood is uncommon mode of transmission of malaria. In endemic regions, the incidence of transfusion-transmitted malaria is under-reported and the incidence varies from zero to two cases per million donations in non-endemic areas (6). There are evidence in the literature suggests that diagnosis of malaria infection have been underestimated.

Here, we report a case of under-diagnosed complicated *vivax* malaria in one-year-old child.

## Case presentation

A one-yr-old girl presented with progressive pallor for six months in Department of Pediatrics, Graduate Institute of Medical Education and Research, Chandigarh, 2016. The child was apparently well six months back when she started having progressive pallor with no fever, bleeding manifestations, pedal edema, cough and rapid breathing. There was no history suggestive of malabsorption, worm infestation, seizure, altered sensorium, jaundice, pain abdomen and decreased urine output. The child was transfused twice outside. There was no history of any chronic illness in the past and antenatal history was uneventful. On examination child was active and playful, brown depigmented hair and pallor was present. There was no icterus, clubbing, edema and lymphadenopathy. On anthropometry examination showed severe wasting (Weight: 6 kg - < z scores) and stunting (Height: 65 cm -< z scores)

On physical examination temperature was 37.8 °C, heart rate 118/min, blood pressure −90/60 mm Hg with respiratory rate of 28/min. The patient was stable, abdomen was distended, hepatomegaly was present and bowel sounds were normal on auscultation. Rest of the systemic examination was within normal limits.

Laboratory examination at admission showed haemoglobin (Hb) – 2.7 g/dl, platelets–290 × 10^3^, MCV–103.2, MCH–31.7, MCHC– 0.7, RDW–19.6; however, the total and differential leucocyte counts were within normal range. Other investigation such as blood and urine culture was sterile, normal hair microscopy and non-reactive HIV serology. Then, the possibility of congenital dyserythropoeitic anemia, bone marrow failure syndrome, schick diamond-menchey clinky hair syndrome and anemia under evaluation was kept and bone marrow biopsy was done.

**Fig. 1:**
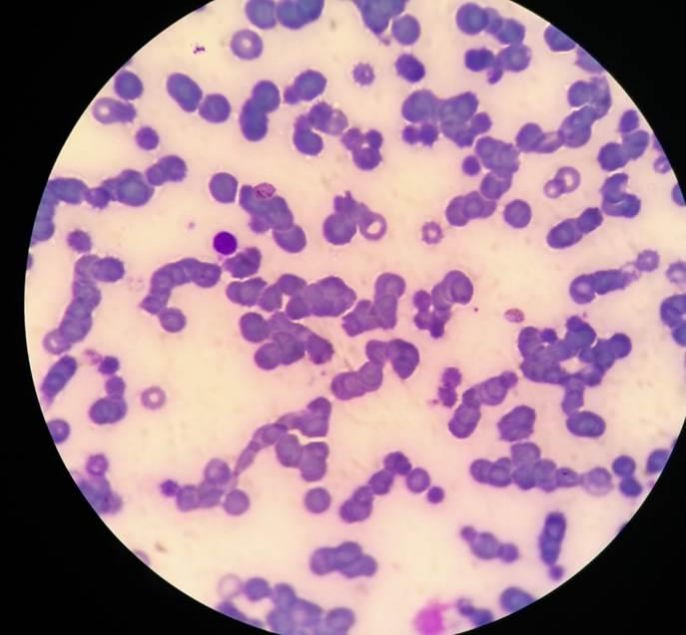
Giemsa stained smear of thin blood smear of *Plasmodium vivax*

Giemsa stained bone marrow biopsy and peripheral blood smear examination showed gametocytes of *Plasmodium vivax* (Fig. 1). Then child was treated with chloroquinine for 3 d, primaquine 2.5 mg ½ once daily for 14 d, folvite 5 mg ½ once daily and blood transfusion was done to correct anemia. Then, haemogram with reticulocyte count and peripheral smear for malaria was repeated after 15 d and peripheral smear was negative for malaria parasites. The child improved symptomatically and doing well.

## Discussion

Malaria is usually transmitted by bite of female anopheles mosquitoes but can also be transmitted through blood transfusion. The most common species in India is *Plasmodium vivax* and *P. falciparum*. The severe complication such as liver dysfunction, kidney dysfunction, anemia, cerebral malaria, multiple organ failure and ARDS is seen in case of malaria infection. Despite representing life threatening condition, transfusion transmitted malaria has gained little attention from researcher worldwide. There is knowledge and research gap regarding transfusion transmitted malaria infection in endemic country like India. The only way to prevent transfusion transmitted malaria infection is screening of blood donors. Early diagnosis and prompt treatment can reduce both morbidity and mortality in complicated malaria infection. Therefore, transfusion transmitted malaria infection needs to be addressed in country moving towards elimination.
